# Supplementary material for: Computational insights and the observation of SiC nanograin assembly: towards 2D silicon carbide
Source: Sci Rep. 2017 Jun 30;7:4399. doi: 10.1038/s41598-017-04683-9 (PMC5493665; doi:10.1038/s41598-017-04683-9)
Supplement: Supplementary file 1 — Supplementary information [file 41598_2017_4683_MOESM1_ESM.pdf]

Supplementary information for

Computational insights and the observation of SiC nanograin assembly: towards 2D silicon carbide

Toma Susi[1,*], Viera Skákalová[1,2], Andreas Mittelberger[1], Peter Kotrusz[3], Martin Hulman[3], Timothy J. Pennycook[1], Clemens Mangler[1], Jani Kotakoski[1], and Jannik C. Meyer[1,*]

[1]University of Vienna, Faculty of Physics, Boltzmanngasse 5, 1090 Vienna, Austria
[2]Slovak University of Technology (STU), Center for Nanodiagnostics, Vazovova 5, 812 43 Bratislava, Slovakia
[3]Danubia NanoTech, Ilkovicova 3, 841 04 Bratislava, Slovakia
[*]toma.susi@univie.ac.at & jannik.meyer@univie.ac.at

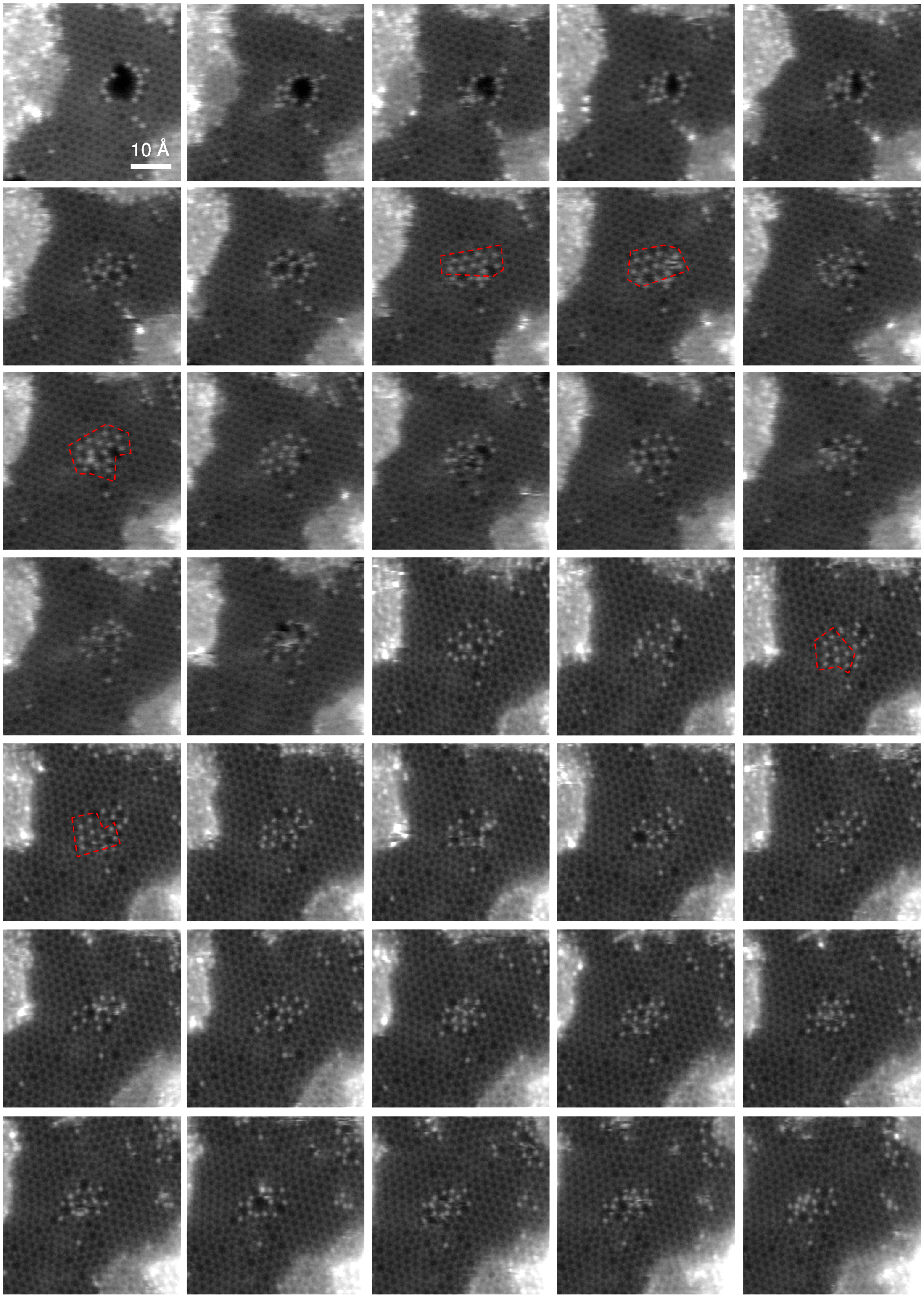

Supplementary Figure 1. An additional time series of SiC grain assembly, with particularly well resolved grains outlined with red dashed lines.

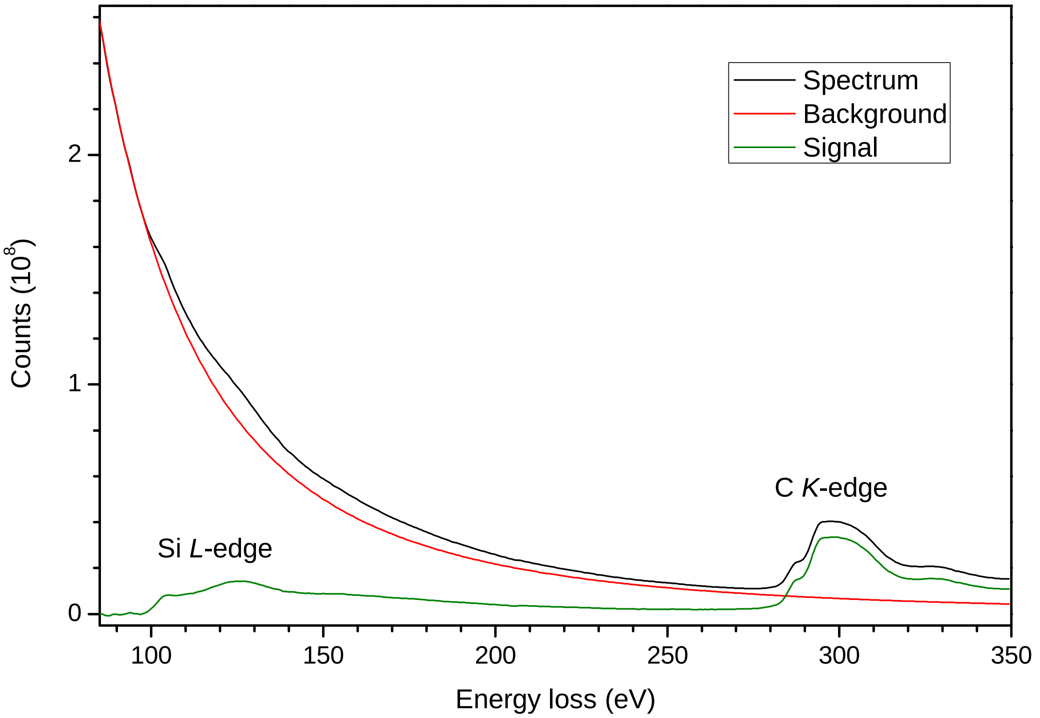

Supplementary Figure 2. An electron energy loss spectrum recorded over the graphene oxide lattice (0.73 eV/px dispersion). The black line is the original spectrum, red is a background fit, and green the resulting signal. Only the C *K*-edge starting at ∼280 eV and the Si *L*-edge starting at ∼99 eV are present in the spectrum.

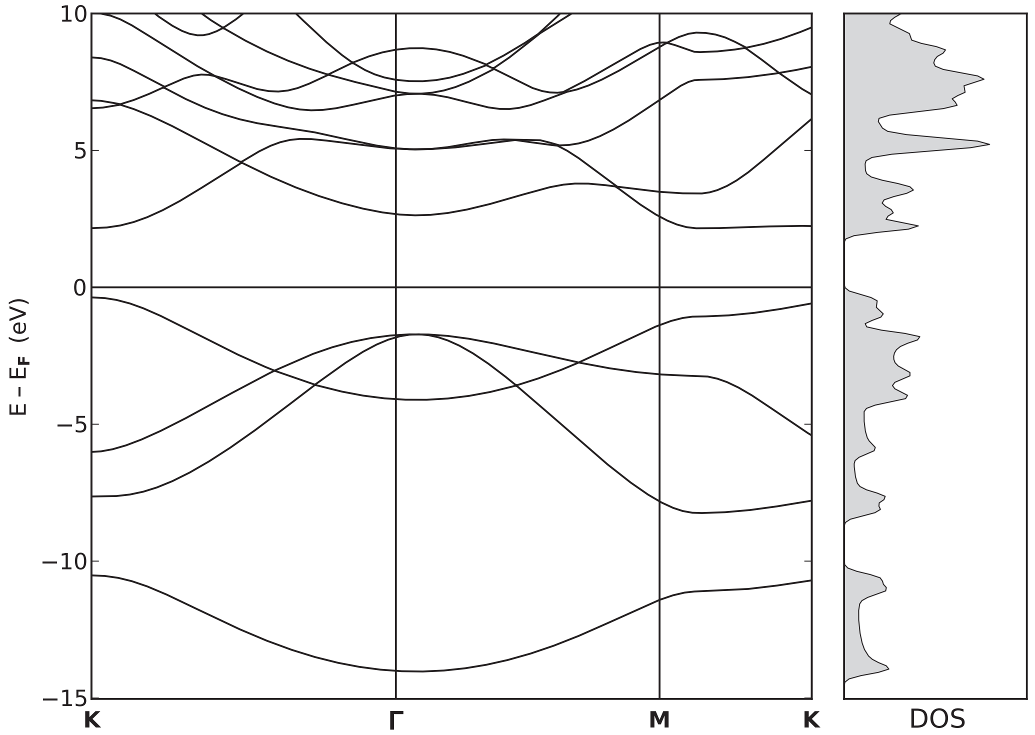

Supplementary Figure 3. The near-gap electronic bandstructure of 2D-SiC calculated with the PBE functional, and the corresponding density of states.

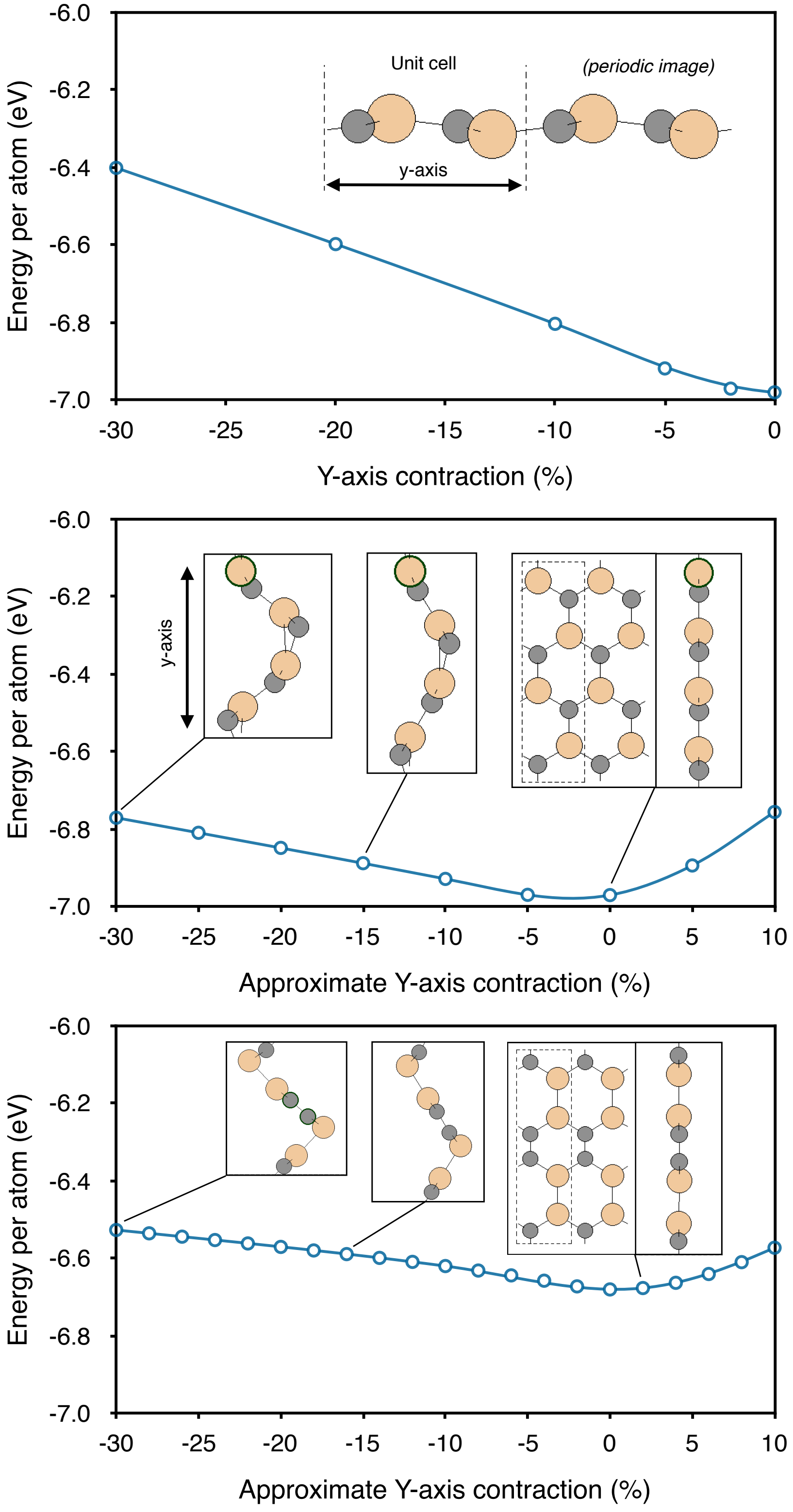

Supplementary Figure 4. Energies of distorted 2D-SiC calculated with density functional theory, demonstrating that the fully flat structure is the ground state. Relaxation of the cell in the perpendicular direction (not shown) does not appreciably change the energies.
